# A Comprehensive Review of Portosystemic Collaterals in Cirrhosis: Historical Aspects, Anatomy, and Classifications

**DOI:** 10.1155/2016/6170243

**Published:** 2016-12-15

**Authors:** Cyriac Abby Philips, Ankur Arora, Rajesh Shetty, Vivek Kasana

**Affiliations:** ^1^Department of Hepatology and Transplant Medicine, Institute of Liver and Biliary Sciences, D-1, Vasant Kunj, New Delhi 110070, India; ^2^Department of Radiodiagnosis, Institute of Liver and Biliary Sciences, D-1, Vasant Kunj, New Delhi 110070, India

## Abstract

Portosystemic collateral formation in cirrhosis plays an important part in events that define the natural history in affected patients. A detailed understanding of collateral anatomy and hemodynamics in cirrhotics is essential to envisage diagnosis, management, and outcomes of portal hypertension. In this review, we provide detailed insights into the historical, anatomical, and hemodynamic aspects to portal hypertension and collateral pathways in cirrhosis with emphasis on the various classification systems.

## 1. Introduction

*Historical Aspects.* Portosystemic pathways form secondary to angioarchitectural changes in the liver in which the blood bypasses an occlusion or a distortion, flowing from high pressure to low pressure areas of vascular beds. Recanalization of embryonic channels and/or reversal of flow in venous systems lead to portosystemic collateral formation [1–3] In 1906, Gilbert and Villaret coined the term “Portal Hypertension” (PHT) to describe a condition characterized by increment in portal pressures, at least 5 mm of Hg above inferior vena caval (IVC) pressure, which was associated with portal circulatory structural changes and gastrointestinal bleeding [4]. The portal venous system and its pathological Roentgen anatomy were initially beautifully delineated by Doenher and colleagues in 1956 [5]. Johns and Evans in 1962 gave the first true anatomical account of natural portosystemic collaterals in a young man who had liver abscess that subsequently lead to development of portal vein (PV) thrombosis [6]. Ruzicka and Rossi demonstrated portal venous pathways using arterioportography techniques in 1969 [7]. The use of computed tomography (CT) for demonstration of caput medusae and paraumbilical collaterals was done by Stanley and colleagues in 1977. Ishikawa and coworkers demonstrated the complete abnormal portosystemic collateral pathway by CT imaging in 1979 and gave descriptions of the anatomical sites of collaterals and compared modalities of CT with that of endoscopy and barium imaging [8]. In 1981, Dokmeci and coworkers evaluated portosystemic collaterals by way of ultrasonography (USG). The standard of diagnosis for collaterals in PHT at the time was splenoportography, abdominal angiography, or percutaneous transhepatic portography which were quite invasive. The frequency of collateral detection using USG was 85% for coronary, 100% for paraumbilical, and 10% for short gastric veins (SGV). The authors concluded that real time sonography needs to be the first-choice procedure in demonstration of collateral veins and diagnosis of portal hypertension [9]. Patriquin et al. in 1987 assessed PHT using qualitative Doppler Sonography and discovered that portal collateral pathways could be easily delineated using techniques that assessed portal blood flow volume, selective flow-velocity measurements, direction, and change in abdominal anatomy. They found that there was a significant association between upper gastrointestinal bleeding and size of left gastric vein (LGV) and esophagogastroscopy was important in such patients, where sclera-therapeutic procedures could be offered [10]. Identification of portal systemic collaterals using blood-spool single photon emission CT (SPECT) was done in 1989 by Kashiwagi and coworkers. The use of direct portosystemic collateral evaluation using scintiphotosplenoportography was found to be inferior to noninvasive use of SPECT in patients of PHT in this study [11]. Computed tomography and angiography could demonstrate more variceal types than endoscopy could and CT was found to be superior to angiography even in detection of paraumbilical and retroperitoneal varices. In 1995, Cho and coworkers improved CT imaging using dynamicity and bolus contrast administration to delineate collateral pathways much better. This modality showed presence of esophageal, paraumbilical, abdominal wall, perisplenic, retrogastric, paraesophageal, omental, retroperitoneal-paravertebral, and mesenteric varices exquisitely and also presence of splenorenal and gastrorenal shunts [12]. In the current review, we provide detailed descriptions on anatomy, classifications, and imaging of portosystemic collaterals in cirrhosis.

## 2. Portosystemic Collaterals in Cirrhosis

### 2.1. Esophageal Varices

**Figure 1:**
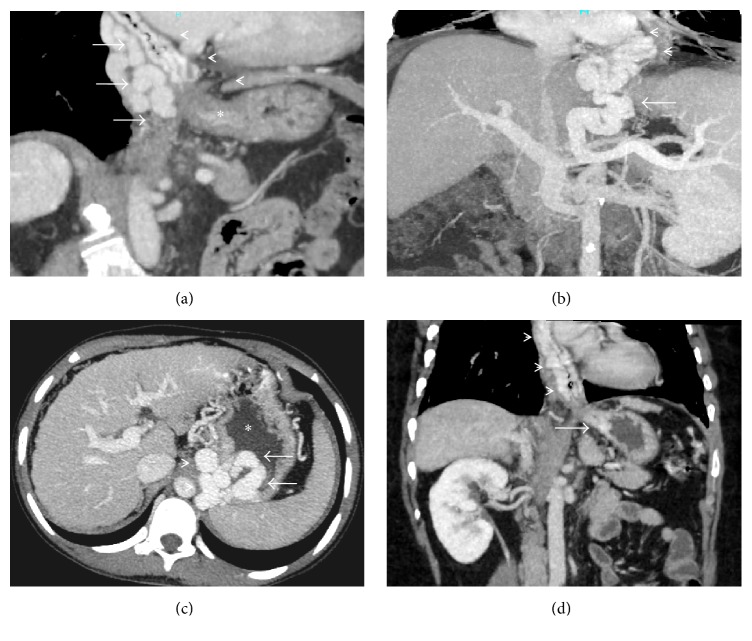
(a) Coronal-oblique MIP image demonstrating multiple collaterals in the esophagus (arrowheads) as well as paraesophageal region (arrow). Asterisk denotes the gastroesophageal junction; (b) coronal-oblique maximum-intensity-projection (MIP) CECT image showing a dilated left gastric vein (arrow) which is serving as an afferent for multiple paraesophageal collaterals (arrowheads); (c) axial MIP image showing multiple gastric fundal collaterals (arrows) being drained by a tortuous gastrorenal shunt (arrowheads) into the left renal vein (not shown). Asterisk denotes the gastric lumen. This corresponds to IGV-1 in Sarin classification of gastric varices; (d) coronal MIP image showing multiple esophageal collaterals (arrowheads) continuing along the cardia to form collaterals in the lesser curvature of stomach. This corresponds to GOV-1 in Sarin classification of gastric varices.

The venous blood from the esophagus drains into the submucosal plexus which in turn drains into the periesophageal venous plexus. From this second plexus, esophageal veins arise in a segmental manner, following the arterial supply. Ultimately the dense submucosal plexus drains into the superior vena cava (SVC) [13]. With regard to esophageal variceal anatomy, 4 distinct zones of venous drainage have been defined around gastroesophageal region: the gastric zone, containing the longitudinal venous distribution; the palisade zone with parallel vessels arranged in groups that lie within the lamina propria; the perforating zone with “treble clef” shaped vasculature which drains blood into the extrinsic veins; and the truncal zone which contains 4 to 5 deep descending veins. This distribution is mainly seen within the esophageal mucosa and the bidirectional venous flow at the palisade zone produces a high resistance water shed area between portal and systemic circulation leading to high accommodation of blood volume in the presence of PHT leading to variceal development [14–16]. Esophageal varices are dilated veins located within lower esophageal wall in contrast to paraesophageal varices that are located outside the wall. The former is usually supplied by the anterior branch of LGV (which dilates >7 mm in diameter in presence of PHT with retrograde flow within) whereas posterior branch takes part in formation of the paraesophageal collateral system. Esophageal varices usually drain into the left subclavian vein and/or brachiocephalic vein, while paraoesophageal varices commonly drain into the azygos or hemiazygos system (Figures 1(a) and 1(b)). Sometimes, only paraesophageal collaterals form without formation of esophageal varices. Portosystemic pathways are normally present embryonic channels that open up in presence of PHT [13]. The common and uncommon portosystemic pathways in cirrhosis and portal hypertension are shown in Table 1 [17–19]. In odd cases, tracheal and bronchial varices also form through efferents of esophageal varices that drain into the bronchial and pulmonary veins connected through bronchial plexus venous system [20].

### 2.2. Gastric Varices

Gastric varices (GV) are defined according to their location and also to their relationship with the esophageal varices. Gastric varices at the hepatofugal collateral pathways can drain into the systemic circulation through two types of collateral systems: the gastrophrenic system or the gastroesophageal system (which eventually drains into the azygous vein). Apart from this, gastric variceal formation in a hepatopetal collateral circulation pathway can develop in case of isolated splenic vein (SV) occlusion. The most commonly used classification is the Sarin classification which does not account for the underlying vascular anatomy. However, the Japanese Society for Portal Hypertension classified GV into cardiac, fundal, or cardiofundal varices which is more refined from an anatomical point of view. In this system, Type 1 gastric (fundal) varices have a single feeding channel that arise from the SV and drain into the left renal vein (LRV) through the gastric cardia and/or fundus and Type 2 gastric varices follow a similar course to the LRV with multiple feeding tributaries [21–24]. In Sarin classification, Type 1 GOV (Figure 1(c)) drains through esophageal and paraesophageal varices, IGV1 (Figure 1(d)) through left inferior phrenic vein, and GOV 2 through both esophageal and inferior phrenic veins. In sinistral (left sided) PHT, in the hepatopetal collateral circulation, drainage is through gastric veins (IGV2). Gastric varices can also drain into azygous vein through the ascending lumbar vein and vertebral plexus of veins. In IGV, the efferent is mainly through the gastric or splenorenal shunt and inferior phrenic vein to the IVC. Gastric varices can also drain from SV to LRV through gonadal vein. Most common drainage routes of IGVs are gastrorenal shunt (GRS; hypertrophied inferior phrenic vein connected to the left renal vein and draining point of left adrenal vein 85%), followed by gastrophrenic shunts (GPS) in 10% and lastly, gastropericardiac shunt in 5%. Another common pathway is the gastrocaval shunt (GCS), where drainage of GV occurs through inferior phrenic or pericardiophrenic vein (which can also drain into the brachiocephalic vein) into the IVC [25–28].

Collateral pathways associated with gastric varices can also be simply divided into those associated with gastroesophageal system or gastrophrenic system. In the former, varices develop between the LGV and azygous system in PHT and in the latter, gastric veins in and around posterosuperior part of gastric wall anastomose with left inferior phrenic vein at the region of gastrophrenic ligament, near the bare area of stomach [29]. In majority of cases (85%) the gastric varices are connected to the SVC through the esophageal varices. Apart from these, the left inferior phrenic vein can also drain into the subcostal, intercostals, right inferior phrenic vein, adrenal vein, or azygous vein thereby providing drainage through these routes also. Isolated gastric varices form due to large portosystemic venous shunts due to anastomoses between gastric vein and left inferior phrenic vein in the presence of PHT [21, 30–33].

## 3. Spontaneous Portosystemic Shunts

**Figure 2:**
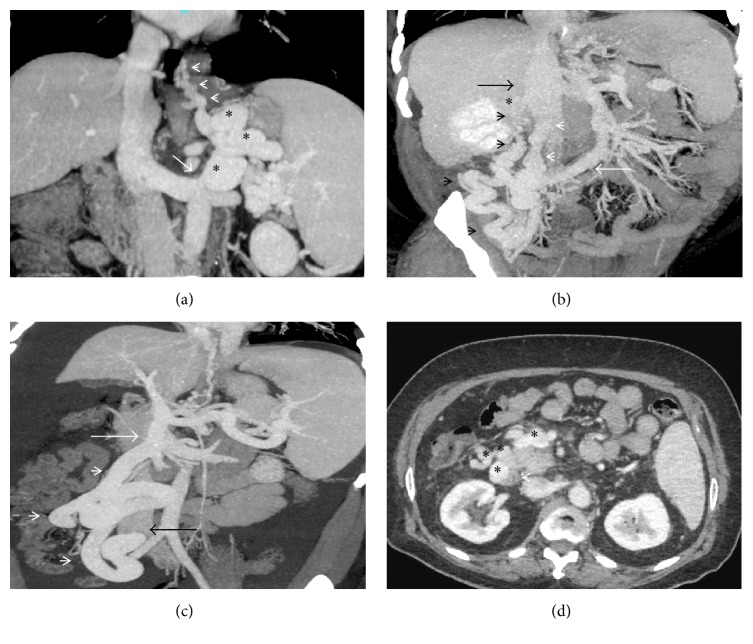
(a) Coronal-oblique MIP image showing a tortuous gastro-lieno-renal shunt (asterisks) draining multiple gastroesophageal collaterals (arrowheads) into the superior aspect of the left renal vein (arrow); (b) coronal MIP image demonstrating a mesentericorenal and a mesentericocaval shunt in the same patient (black and white arrowheads, resp.). Asterisk: right renal vein, black arrow: IVC, and white arrow: superior mesenteric vein (double portosystemic shunts); (c) coronal-oblique MIP image demonstrating a dilated and tortuous mesentericocaval shunt (arrowheads) communicating between the superior mesenteric vein (white arrow) and the inferior vena cava (black arrow); (d) axial CECT image showing multiple duodenal and paraduodenal collaterals (asterisks). Arrowhead denotes the duodenum.

Spontaneous portosystemic shunts (SPSS, Figures 2(a)–2(c)) develop between the portal and systemic venous circulation and grow in relevance to enable large amounts of flow within them. This flow stabilizes and increases when either the portal or systemic venous circulations develop high pressure or is obstructed or both occur. Spontaneous shunts develop in an attempt to reduce high pressures or bypass an obstruction.

Spontaneous portosystemic shunts can be divided into left and right (or central) sided shunts. Left sided shunts are described based on their location (left of midline or left of the confluence of splenic and mesenteric vein). These shunts have hemodynamic involvement directly or indirectly with the SV or its branches, namely, the LGV, SGV, or PGVs. Left sided SPSS include the GRS occurring in 80 to 85% of patients in gastric varices, GCS, the least frequent type of left sided PSS, but associated with gastric varices and SRS which is not uncommon in cirrhosis patients and has no association with gastric varices. Gastrorenal shunts occur in 10% of patients with PHT. The communication is between GV and LRV, but in reality, it is a small part of the larger PSS communication that involves the SV along with LRV and GV. Hence, hemodynamically, this is a SRS. This whole complex is termed the gastric variceal system. Hence, the part of this complex, which encompasses the GRS, should be ideally called the splenogastrorenal shunt.

Splenorenal shunt is not in true terms a hemodynamically described shunt. This shunt is more likely a morphologic or anatomical shunt and is not associated with gastric varices. It is tortuous, meandering direct communication between SV and the LRV without involvement of the gastrointestinal tract and is a vascular channel without variceal formation and without spontaneous bleeding risk.

## 4. Descriptive Classifications of Gastric Variceal System and Gastric Varices and Importance of Portosystemic Shunts

The gastric variceal system includes GRS and central part of GV with or without the afferent portal venous feeding collaterals. It is important to keep in mind that GRS is hemodynamically a part of a larger complex that is SRS and that morphologically the SRS is not associated with GV (absence of shunt extension into the gastric wall). It is also important to note that gastric varices may have a direct component that is part of the SRS but does not contribute to gastric varices formation, but, in reality, bypassing the stomach wall.

**Figure 3:**
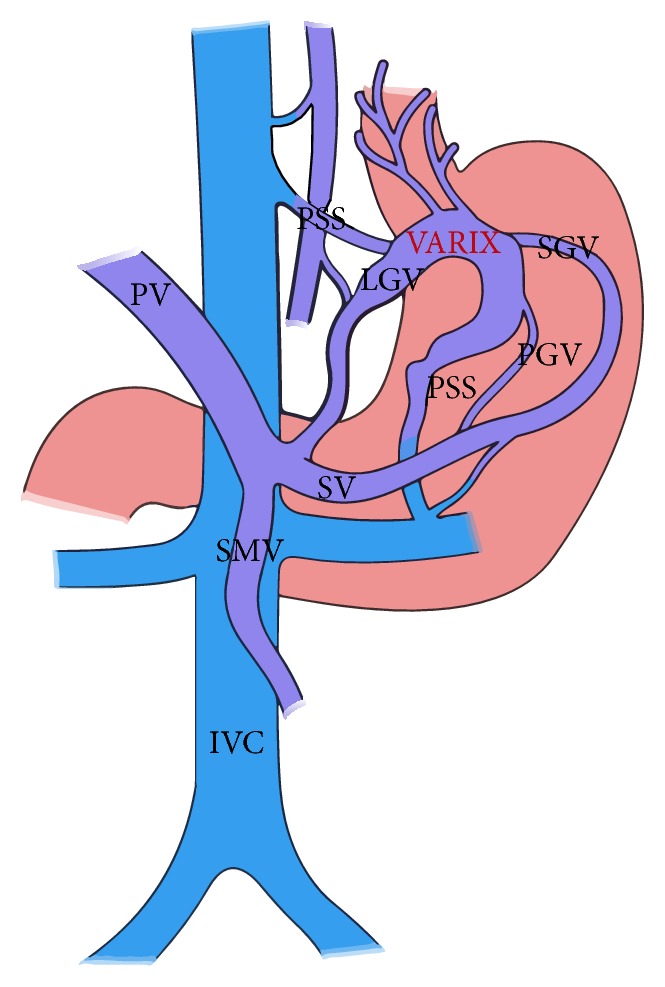
The basic portosystemic venous anatomy of gastric varices. PV: portal vein, SV: splenic vein, SMV: superior mesenteric vein, IVC: inferior vena cava, PSS: portosystemic shunt, SGV: short gastric vein, LGV: left gastric vein, and PGV: posterior gastric vein. Modified from [34].

The GV have an afferent limb (portal inflow), central part, and efferent limb (systemic venous outflow) portions. The portal inflow or afferent feeders supply GV. These feeders do not directly communicate with true GV (intragastric, submucosal) but do so with GVs outside the gastric wall and form the extragastric or false GVs (Figure 3).

The feeders include LGV (or coronary vein), PGV, and SGVs. The PGV, which is usually single, can have a duplication and early bifurcation leading to multiple feeder profile, making it difficult to identify a laterally lying PGV from a medially lying SGV that could be acting as an afferent and is an important technical implication in interventional treatment.

Left gastric vein is the right sided component of SPSS because it comes off from the PV near the midline. Posterior and SGVs are also left sided parts of the portosystemic system. The SGVs traverse forward medially towards the GVs from the splenic hilum and enter the varix at a level higher, anterior or ventral, than the PGVs. The dominance of portal inflow feeders is important. In some, the dominant feeder is the LGV, but PGV or SGVs can also act dominant. If the gastric variceal system is very complex, then all three feeders become equally dominant. The only condition in which the SGVs become the only dominant feeder is in presence of SV or PV thrombosis. If SGVs become the dominant feeder in portal hypertensive gastric variceal complex formation, the gastric varices extend over fundus, cardia, and also body, antrum, and gastric outlets. The central part of the gastric variceal system consists of true submucosal intragastric and false extragastric varices. Sometimes these also communicate with each other through a singular perforator vein. Gastric varices are also seen between prominent folds in the stomach, but they can also be confused with prominent gastric rugal folds or self-thrombosed GVs. Regarding extragastric varices that do not have an intragastric submucosal component, the afferent feeders usually drain into these and perforators commence from them to penetrate the gastric wall to form true GVs. The efferent or outflow system of GVs may be simple (only GRS) but can also be very complex, involving the inferior phrenic or pericardiophrenic veins. To understand the outflow system, it is important to understand the GRS and the pericardiophrenic collateral pathways. The GRS commences from false gastric (extragastric) varices and starts as small convolutions intraperitoneally in the lesser sac. It travels caudally and posteriorly and becomes retroperitoneal as the flow increases and it grows in size. This initial convoluted portion is termed the intraperitoneal transition zone. In the retroperitoneal region, the shunt becomes less convoluted and reaches the left adrenal vein (area of common stump). The entrance of the retroperitoneal portion of the shunt with the left adrenal vein occurs at different angles. The commonest angular relation of the shunt with the stump is between 10 and 12 o' clock positions. Less commonly, the angle between the shunt and the stump occurs at 12 and 1 o' clock positions. In rare situations, the retroperitoneal portion of the shunt joins the left gonadal vein instead of the left adrenal vein. More in rarity is the duplication of the GRS in which the LRV supply the shunt, forming anterior and posterior GRS. The phrenic vein or inferior phrenic vein has two parts, the infradiaphragmatic horizontal position and the vertical part (composed of ascending and descending regions). It is the descending part that forms a component of GRS by anastomosing with the left adrenal or left gonadal vein. The phrenic venous system can become complex in the presence of duplication, ecstatic trunk, or multiple isolated veins which then form a complicated system of collaterals. The pericardial vein traverses the left heart border and after travelling along the left hemidiaphragm, it anastomoses with the transverse part of inferior phrenic vein. The pericardial vein and the inferior phrenic vein are collectively called the pericardiophrenic vein [21, 36, 37].

## 5. Hemodynamic Classification of Gastric Variceal Systems

The new classification systems for GVs are descriptive for hemodynamics (based on efferent inflow or afferent outflow) of variceal system rather than just their location. These include the Saad-Caldwell Classification for gastric varices (inflow and outflow) and the Saad Classification for gastroduodenal and mesenteric variceal systems (dealt with later on, under ectopic varices). Also important are the Kiyosue Efferent Classification System, Hirota-BORV Classification, Fukuda-Hirota Classification, and the Matsumoto Hemodynamic Classification Systems.

### 5.1. Classifications Based on Draining Venous Anatomy (Efferent Outflow)

**Figure 4:**
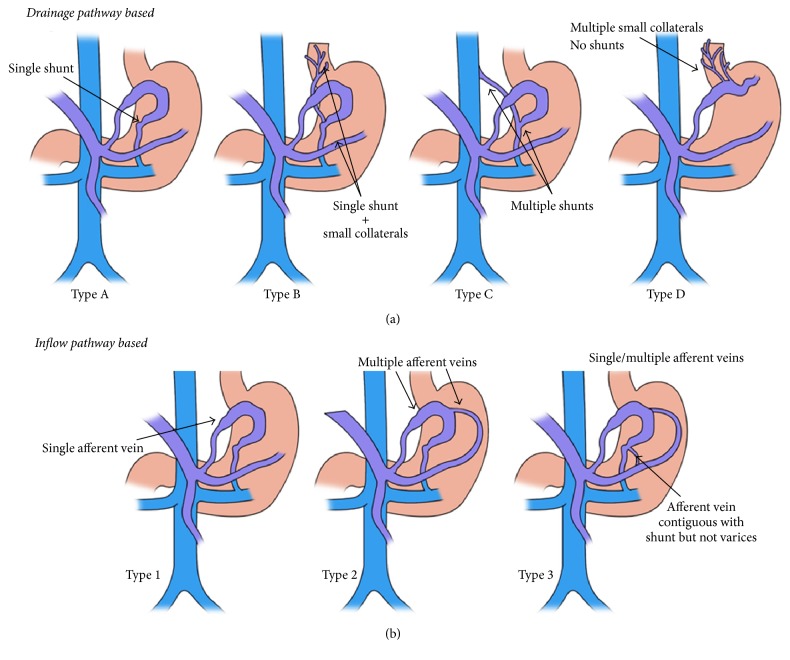
The Kiyosue classification of gastric varices. (a) Classification based on drainage pathway. Type A consists of a portosystemic shunt as the only drainage; Type B portosystemic shunts along with additional small portosystemic collaterals; in Type C, there is presence of multiple large portosystemic shunts; and Type D consists of multiple small portosystemic collaterals as the drainage pathways without proper shunt formation. (b) Classification based on the inflow pathway: Type 1 consists of single afferent vein for the varices; Type 2 has multiple afferent vessels contributing to the variceal formation; Type 3 is similar to Type 2 but with additional small collateral/shunts directly communicating with outflow tract. Modified and redrawn from [17].

- *The Kiyosue classification of gastric variceal system* (Figure 4(a)) is a simple way based on hemodynamic classification. In this classification, they are divided into 4 types. In Type A, the varices are in continuation with a single draining shunt (most commonly a GRS shunt or, seldom, GCS) draining through hypertrophied inferior phrenic vein into IVC directly. In Type B, the GVs are contiguous commonly with a GRS and one or multiple collateral veins. This drains through a plexus of vessels back into the right atrium or IVC but there is no distinguished shunt formation. The draining veins can include pericardiophrenic, intercostal, perivertebral, ascending lumbar, and, rarely, azygous veins. Type B is further divided into three types: B1, small collateral veins; B2, medium sized collateral veins; and B3, large collateral veins with high flow but without shunt. Type C varices are contiguous with both GRS and GCS and this is also subdivided into C1, representing small sized second shunt that cannot be catheterized, and C2, large sized second shunt which is large enough to be catheterized. Finally in Type D, a shunt is not present and the varices drain through small collaterals and do not drain directly into IVC or renal vein.
- In the original* Saad Classification* the characterization of collaterals and shunts is similar to that in Kiyosue, except for subclassification in Type D, as Types D1 and D2. In Type D1 of Saad modification, the predominance of systemic vein drainage is not obvious and any vein, out of inferior phrenic, hemiazygos tributaries, and intercostals veins or adrenal veins, can become predominant. In Type D2, the morphology is similar to D1, but predominant systemic venous draining vein is usually 4.3 mm in diameter through unconventional systemic veins.
- *The Hirota Classification* follows similar descriptions as the Kiyosue classification (for efferent pathway), except for Hirota-BORV Type V in which a gastrorenal shunt is too large for balloon occlusion procedures (no catheter size available) and maybe a cause of technical failure during BRTO treatment. For such shunts, Balloon Antegrade Transvenous Occlusion has been proposed.

### 5.2. Classifications Based on Portal Afferent Inflow

- Based on portal venous* afferent inflow* (Figure 4(b)),* according to Kiyosue*, gastric varices are classified into three types, with regard to pattern of afferent veins. In Type 1, the varices are supplied by a single afferent gastric vein, which is the commonest and easiest type to treat. In Type 2, multiple afferent gastric veins supply the varices and in Type 3, single or multiple gastric veins supply the varices in presence of other gastric veins that are directly in continuation with a shunt, which do not significantly contribute to variceal formation. In Type 1, the afferent is most commonly the LGV or PGV. In Type 2, it is both LGV and PGV and in Type 3, a separate afferent vein drains directly into a shunt that does not communicate with the GVs [38, 39]. Clinical implications of Types 1 to 3 GVs with regard to venous inflow are that, because of simplicity of anatomy in Type 1, during occlusion procedures of collateral pathways, the administered sclerosant refluxes into the gastric variceal complex and stays there because of high pressure from the portal system allowing for minimal reflux into afferent vein and better chances at complete occlusion. In Type 2, since two pathways act simultaneously, one of the low pressure efferent veins functions as a draining vein once outflow is obstructed with sclerosant, resulting in efflux of material into portal circulation. In Type 3, since the separate vein does not supply the GVs, during occlusion procedures, the sclerosant can reflux into the portal circulation rather than the gastric variceal complex. Hence, separate embolization of this noncommunicating shunt will be required for complete variceal occlusion [40–45].
- *The Saad-Caldwell Classification* (Table 2) can be used uniformly in all splanchnic varices (i.e., gastric duodenal and mesenteric). It is mainly applicable in the management of duodenal and mesenteric varices. Saad modified the gastroesophageal varices system proposed by Al-Osaimi and Caldwell which was a revamped version of Sarin classification of gastric varices, to include management modality. In Saad Type 1, the dominant portal venous feeder is the left gastric vein, commencing from either the main PV, splenoportal junction, or the distal SV. This can be associated with presence (Type 1b) or absence (Type 1a) of gastrorenal shunt. In Type 2, the dominant portal venous feeder is the PGVs or SGVs. In Saad Type 3 all feeders that are involved have dominance that is variable. Type 4 Saad gastric varices are more similar to Type 3 but occur in the presence of SV thrombosis [46, 47].

### 5.3. Other Hemodynamics Based Classifications of Gastric Variceal Systems

- *Hirota* and coworkers classified GVs based on balloon retrograde transvenography (BRTO). They classified the degree of progression of gastric varices and collateral veins into five grades: Grade 1: gastric varices well opacified without any collateral vein evidence; Grade 2: contrast opacification in gastric varices for ≥3 minutes, in the presence of small and few collateral veins; Grade 3: contrast opacification of gastric varices partial and disappeared within 3 minutes with medium to large collateral veins which were few in number; Grade 4: noncontrast opacification of gastric varices and presence of many large collaterals; and Grade 5: in which shunt could not be occluded because of very large size of shunt and rapid blood flow [48].
- *The Fukuda Classification System* of gastric varices is different from the Hirota Classification [49] because it is based on hemodynamic features involving the superior mesenteric and celiac angiography findings. In this classification, Type 1 refers to LGV dominant gastric variceal complex and Type 2 refers to the separation between the esophageal varices (LGV dominant) and the GVs (PGV/SGV dominant). In Type 4, there is right sided dominance of gastric variceal system and Type 3 is a very complex system consisting of both right and left sided feeding vessels.
- *Matsumoto* et al. described a* classification system* for gastric varices for predicting the aggravation of esophageal varices after BRTO procedure [50]. This is based on left gastric angiography. The classification includes Type 1 which has portosystemic flow in the gastrorenal shunt and Type 2 which has no portosystemic flow in the gastrorenal shunt. In both, subtypes have been described, in which Subtype A has hepatopetal flow and Subtype B has hepatofugal flow in the LGV. Aggravation of grade of esophageal varices occurred after BRTO in Type 1b varices, which had portosystemic flow in the GRS (the occlusion of shunt causes back flow and worsening of variceal grade).

Gastric varices can also be differentiated as* primary* (naturally developed) and* secondary* (developing after endotherapy for esophageal varices, seen in 9%) [51].

## 6. Ectopic Varices

**Figure 5:**
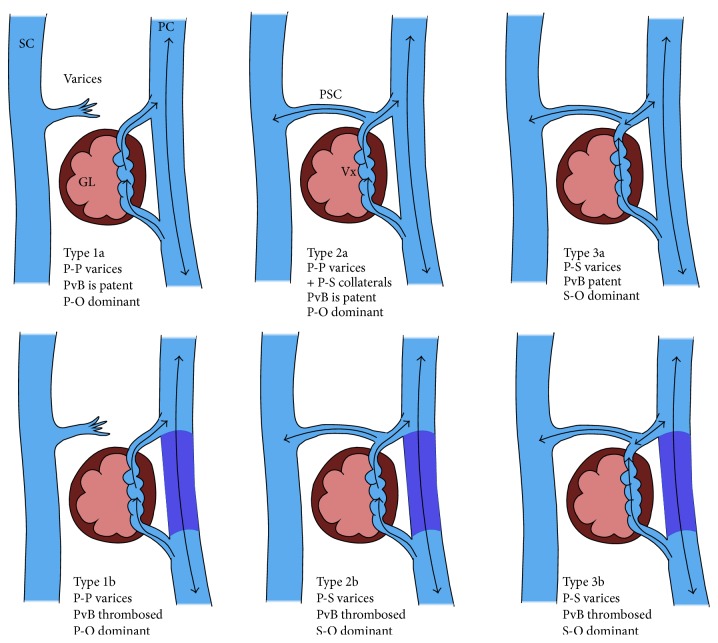
Classification of ectopic varices. SC: systemic circulation; PC: portal circulation; P-P: portoportal collaterals, P-S: portosystemic collaterals; PvB: portal venous branch; P-O: portal outflow; S-O: systemic outflow. Modified and redrawn from [35].

These are dilated splanchnic or mesoportal varicosities and/or portosystemic collaterals that are present along the gastrointestinal tract outside of the common variceal sites. These can be broadly divided into Type a (nonocclusive or oncotic) and Type b (occlusive) subdivided into Type 1 (purely portoportal collaterals), Type 2 (predominantly portoportal collaterals with some portosystemic branches), and Type 3 (predominantly portosystemic collaterals with some portoportal branches) as per Saad-Caldwell Classification (Figure 5). The portal venous branch can be any vein (location or size) in the portal circulation which includes mesenteric vein and tributaries and PV tributaries as well as the main portal and splenic veins. The importance of this classification lies mainly from therapy point of view. For example, balloon-occluded retrograde transvenous obliteration (BRTO) of Type 1 varices is not feasible because, by definition, BRTO is through the portosystemic collaterals from the systemic venous side and in Type 1, there are no portosystemic collaterals. Hence, obliteration from the portal venous side (BATO: balloon-occluded antegrade transvenous obliteration) is more feasible in this scenario.

## 7. Duodenal Varices (Figure 2(d))

The first 2.5 cm (duodenal bulb) is drained by the prepyloric vein which drains into the PV and the rest of duodenum is drained by the superior pancreaticoduodenal veins that drain into the PV and the inferior pancreaticoduodenal veins that drain into the SMV (which is present to the right of superior mesenteric artery in front of third part of duodenum). The pancreaticoduodenal veins are four small veins that also drain the head of pancreas and adjacent second and third parts of the duodenum, forming a venous arcade (anterior and posterior) between the superior and inferior veins. It is the posterior superior pancreaticoduodenal vein that drains into the PV and the anterior superior pancreaticoduodenal and posterior inferior pancreaticoduodenal veins that drain into superior mesenteric vein. Duodenal varices (DV) were first reported in 1931 by Alberti. Duodenal variceal bleeding represents only 1/3rd of all ectopic variceal bleeding sources, even though in some studies, the prevalence of paraduodenal varices by angiography demonstration was as high as 40% [52]. Duodenal varices make up 1 to 3% of all varices in patients of PHT. These varices are smaller in diameter and shorter in length and found in deeper locations or over paraduodenal areas and enter through the perforators in the submucosal region. Duodenal varices can occur on the serosal surface as well as in the muscular layer, but bleeding manifestation is evident only when they expand onto the submucosal surface. In a study by Amin and coworkers, the commonest site of DV was found to be at the duodenal bulb [53]. There have been studies on association of DVs with GEVs and it has been shown in two different studies that DV were more commonly associated with intrahepatic as well as extrahepatic PHT [54]. Further studies on this association were confirmed in a study by Stephan and colleagues wherein they found that 40% of patients had an extrahepatic source of PHT [55]. Unlike colonic and jejunal varices, DVs have never been associated with previous surgical adhesions and it has also been shown to be associated with postsclerotherapy for esophageal varices.

Duodenal varices are portoportal or portosystemic retroperitoneal collaterals or a combination of both (see classification of ectopic varices below). The afferent (portal venous feeders) includes inferior and superior pancreaticoduodenal veins, cystic branches of superior mesenteric veins, pyloric vein, and gastroduodenal vein. The efferent or systemic drainage channels include gonadal vein (mostly the right) and capsular renal veins that ultimately drain into the inferior vena cava. The left gonadal vein, when involved, produces ectopic duodenal varices at third and fourth parts of the duodenum and is very rare. Rarely, direct drainage into inferior vena cava is also seen as is efferent drainage through paravertebral or innominate retroperitoneal veins. The pathways associated with DV differ in cirrhosis and in patients with extrahepatic portal hypertension. In cirrhosis, the efferents are formed in descending or transverse parts of duodenum, flowing hepatofugal through retroperitoneal veins of Retzius (small shunts), finally into the IVC and also through subcostal and ascending lumbar vein into vertebral-lumbar azygous system and ultimately draining into the SVC. In patients with extrahepatic portal hypertension (commonest cause of duodenal varices), the efferents are formed mostly in first 2.5 cm of the duodenum (bulb region) with a blood flow that is hepatopetal through portoportal collaterals into liver. These portoportal collaterals form from venous plexus around common bile duct or tributaries of PV that are patent above obstructed portion of the portal venous system. Next most common site of duodenal varices is the second part of duodenum and rarely in third and fourth parts [53, 55].

## 8. Varices of Jejunum and Ileum (Figure 6(a))

The jejunal and ileal veins eventually drain into the SMV which drains into the IVC through the pelvic or retroperitoneal veins. There are many veins on the dorsal wall of the abdomen, called veins of Retzius that form anastomosis between the IVC and the SMV/IMV. This anastomosis can occur normally in the absence of PHT. The veins of Retzius group gives rise to various portosystemic pathways, such as mesenteric-caval, mesenteric-iliac, mesenteric-gonadal, or mesenteric-renal. Out of these, the commonest is an ileocolic vein draining into IVC or the right renal vein by way of the right gonadal vein, the mesenteric-gonadal pathway. Jejunoileal varices develop mainly at sites of prior surgeries or adhesions after surgery, mostly between the jejunum and the abdominal wall. The afferents of jejunoileal varices are mainly the tributaries of the superior mesenteric vein and the efferents are veins of the abdominal wall and also through the veins of Retzius [56–58].

## 9. Varices of the Colon (Figure 6(b))

The venous supply of colon mirrors arterial supply. The ileocolic, right (draining ascending colon), and middle colic veins (draining transverse colon) are tributaries of the SMV and the superior and inferior left colic veins are tributaries of inferior mesenteric vein. The IMV drains into the SV and the SMV joins the SV to form the PV. The cecum is drained by the anterior and posterior cecal veins which arise from the ileocolic veins which are tributaries of the SMV. Colonic ectopic varices are normally seen in the cecal and rectosigmoid regions, found in a segmental distribution. The afferents to colonic varices include ileocolic vein, right colic and middle colic vein, and sigmoid colic vein while the efferents include right gonadal vein, right renal vein, and systemic lumbar veins that drain into the veins of Retzius and veins of the ascending colon that drain through renal capsular vein into IVC [59–61].

## 10. Varices of Rectum and Anal Canal (Figure 6(c))

The pectinate line (or dentate or mucocutaneous line) is an important landmark in defining certain anatomic points in the rectum and anal canal anatomy. It is present at the inferior most level of anal columns and defines the junction of superior part of anal canal and the inferior part of anal canal, both of which have different embryonic origins. The vascular supply to rectum and anal canal is defined by making pectinate line a landmark. The superior rectal vein divides into two branches, which enter the lateral wall of rectum, about 10 cm above the dentate line. The middle and inferior rectal veins drain into the caval system. The rectal veins form two plexuses, one lying in the submucosa (intrinsic) and the other lying outside the muscular wall of the bowel below the level of the peritoneal reflection (extrinsic). Above the pectinate line, the intrinsic rectal plexus drains into the superior rectal vein and below the pectinate line it drains into the inferior rectal veins. The intrinsic rectal venous plexus consists of two groups of veins draining in opposite direction. The inferior group passes down to form the inferior rectal veins, dilation of which leads to formation of external hemorrhoids. The vessels of the superior group in the anal columns dilate to form internal hemorrhoids and those in the rectum form rectal varices. Rectal varices (RV) were first reported in 1954. Rectal varices are dilated submucosal portosystemic collaterals that extend from midrectum to the anorectal junction and are considered distinct from internal hemorrhoids (submucosal arteriovenous communications of the anorectal vascular plexus). Four distinct zones of mucosal circulation similar to those seen in esophagus occur in the rectum also: an inflow area analogous to the gastric zone, the downflow area analogous to the palisade zone, outflow area in the rectum which is analogous to the perforating zone, and the outflow area in anal canal which is analogous to the truncal zone of esophageal varices. The submucosal portosystemic communications of rectal varices have hepatofugal inflow. The flow to the intrinsic rectal venous plexus occurs through the wall of rectum by branches of superior rectal vein, a tributary of inferior mesenteric vein.

From both the plexuses, the portal hemorrhoidal blood drains into systemic circulation through two portosystemic shunts (rectogenital and interrectal). The rectogenital communication connects the rectal venous plexus with vesicoprostatic or vaginal venous plexus. Theinterrectal communications occur between the three rectal veins. Hemorrhoids occur independent of rectal varices. In a large series of cirrhotics, it was shown that RV were present in 44% and bleeding from rectal varices occurs in 8% and that the prevalence increased with increase in severity of PHT: 19% more in patients with cirrhosis and without esophageal varices, 39% in patients with esophageal without prior bleeding, and 59% in patients with prior bleeding. Thirty percent of patients can have concomitant hemorrhoids with RV and it is very important to differentiate rectal varices from hemorrhoids.  Table 3 shows important characteristics of RV and their differentiating points from hemorrhoids [62–64].

## 11. Biliary Varices and Portal Hypertensive Biliopathy (Figures 6(d) and 7(a))

These are most commonly seen in extrahepatic obstruction of PV (EHPVO) and can cause extensive collateral venous circulation at porta hepatis. The description of portal biliopathy was first given in 1944 by Fraser and later on by Gibson in 1965. The term portal biliopathy or pseudosclerosing cholangitis was first coined in early 90s and used to describe abnormalities of extra- and intrahepatic biliary tract, gall bladder, and cystic ducts in patients of PHT. Even though biliary tract changes occur in 80 to 100% of EHPVO patients, symptomatic obstruction occurs in only 5 to 30% patients. Anatomical understanding of varices in the biliary tract stems from pristine description provided by Petren and Saint in 1932 and 1971, respectively. There are two venous systems that run along the biliary tract: the paracholedochal veins (of Petren) that run parallel to the common bile duct and the epicholedochal plexus (of Saint) forming a fine reticular network over the common bile duct. The former is a separate system from the bile duct, so much that variceal formation within this group produces extrinsic compression whereas dilatation of the latter group causes only mild to moderate irregularities of the biliary ductal system. The venous plexus of Petren is connected with the gastric, pancreaticoduodenal, and portal venous system and also to the liver directly. The right sided biliary plexus communicates with gastrocolic vein and pancreaticoduodenal vein to cystic vein or directly to liver. Left side biliary venous plexus communicates with jejunal veins, left and right gastric vein, and the left PV with flow moving towards branches of PV [65, 66]. Endoscopic retrograde cholangiography (ERCP) and endoscopic ultrasonography have helped in identifying these biliary varices and map their anatomy well. On ERCP, the biliary abnormalities secondary to biliary varices include smooth strictures, luminal irregularity, segmental dilations, indentations, ectasias, angular deviations of ducts, and pruning and clustering of intrahepatic ducts. Left hepatic duct is more involved than the right probably because collateral veins are more frequently formed between the paraumbilical veins and the left branch of PV compared to the right system. ERCP grading of biliary varices/portal biliopathy has been divided into 4 types: Type 1 in which there is involvement of extrahepatic bile duct only, Type 2 with intrahepatic bile ducts only, Type 3a with extrahepatic and unilateral intrahepatic bile duct involvement, and Type 3b with extrahepatic and bilateral intrahepatic ductal involvement [67, 68]. On EUS evaluation, the collaterals enter subepithelial layer of CBD after perforating the fibromuscular layer of CBD wall forming intracholedochal varices. Perforators from the paracholedochal collaterals act as link between veins outside and inside the muscular wall of CBD. Currently EUS could be the investigation of choice in evaluation of origin, caliber, entry, and course of intracholedochal varices throughout the CBD. Early on, changes occur in the paracholedochal vein of Petren which later involve those of Saint [69–72].

## 12. Other Ectopic Varices and Their Afferent and Efferent Pathways Including Veins of Sappey and Burrow: Umbilical and Paraumbilical Veins in Portosystemic Collaterals

**Figure 7:**
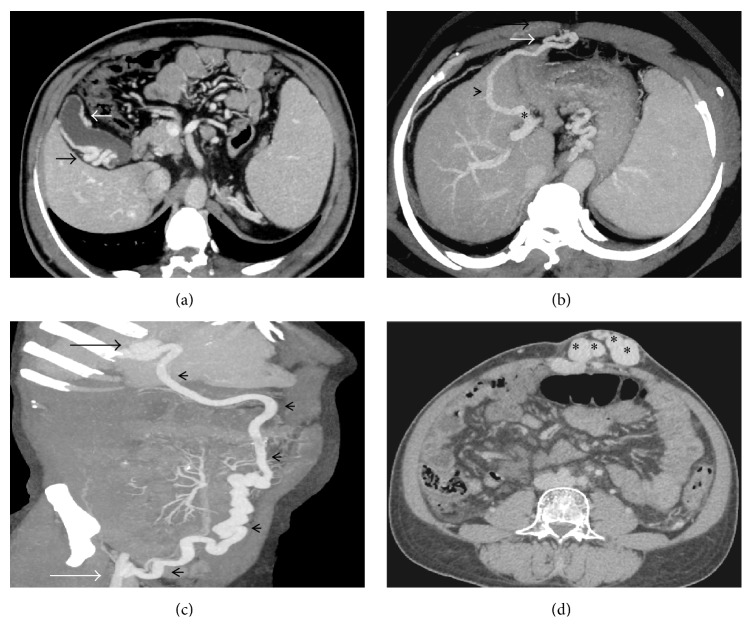
(a) Axial CECT image showing multiple pericholecystic collaterals (arrows); (b) axial-oblique MIP image showing a right infradiaphragmatic type of shunt (arrowhead) arising from the left portal vein branch (asterisk) and draining into the intercostal vein; (c) coronal-oblique MIP image demonstrating a prominent recanalized paraumbilical vein (arrowheads) arising from the left branch of portal vein (black arrow) and draining into the right internal iliac vein (white arrow); (d) caput medusa, multiple periumbilical abdominal wall varices (asterisks).

Burrow in 1838 described a pair of veins ascending from inferior epigastric veins along the umbilical vein and uniting into a single channel, draining into the upper part of umbilical vein. Sappey in 1883 noted that paraumbilical veins were distended in patients of PHT on postmortem studies. He referred to these veins as accessory PVs, which had superior and inferior divisions. The superior group drains the median part of diaphragm and traverses the falciform ligament in its upper part to reach the convex surface of liver, entering the sublobular branches of PV. The tributaries of the inferior group run alongside inferior part of falciform ligament, entering the hepatic fissure. Inferiorly they communicate with epigastric and cutaneous veins and become dilated in presence of PHT: one channel gets selectively dilated to connect the right epigastric vein with the liver [73, 74]. Later on, it was shown that umbilical vein and the veins of Burrow also become dilated in the presence of PHT and that the dilated veins of Burrow communicate with dilated deep epigastric veins after piercing the rectus sheath. Veins of Burrow can also enter the portal system directly and correspond to the inferior veins of Sappey. On the contrary, Sappey's veins when they drain into the umbilical vein came to be known as intercalary veins of Baumgarten. Sappey's veins enter the portal system through the liver capsule from different directions and form transhepatic portosystemic shunts in the liver. The common locations of occurrence of Sappey's veins are shown in the following list. The superior veins of Sappey connect the convex anterosuperior part of the liver surface to the diaphragm and connect with internal thoracic veins. The paraumbilical veins (inferior veins of Sappey) in PHT connect the anterior parietal veins like the superior and inferior epigastric veins in rectus sheath and thoracoepigastric vein in subcutaneous tissue at the umbilicus with the left branch of PV. These connections can also occur in the anterior abdominal wall, around the umbilical regions, forming “caput medusae.” Portosystemic collaterals also occur through the bare area of liver (right inferior phrenic vein) and the left triangular ligament (through left intercostal, pericardiophrenic, and inferior phrenic veins). Caput medusae is also responsible for varicosities in the anterior right thigh and is also associated with varicose veins in legs [75–77] (Figures 7(b)–7(d)).

Sappey's veins and their anatomical considerations are as follows:

Upper and lower part of falciform ligament.
Left triangular ligament: left inferior phrenic vein and intercostal vein regions.
Ligamentum teres, central portion of falciform ligament, and recanalized umbilical vein.
Right triangular ligament: right inferior phrenic vein region.
Diaphragmatic veins (near bare area of liver).
Ligamentum venosum: patent ductus venosus.
Gastrohepatic omentum: cystic veins and branches of left gastric vein region.

## 13. Conclusions

The current review describes various portosystemic collateral pathways pertinent to portal hypertension and their current classifications beyond traditional, with special emphasis on hemodynamics. It is very important that the Hepatologist and the interventional Radiologist know and understand the various patterns of portosystemic collateral channels for accurate diagnosis, sensible management, and prevention of accidental vascular injury during intervention.

## Figures and Tables

**Figure 6:**
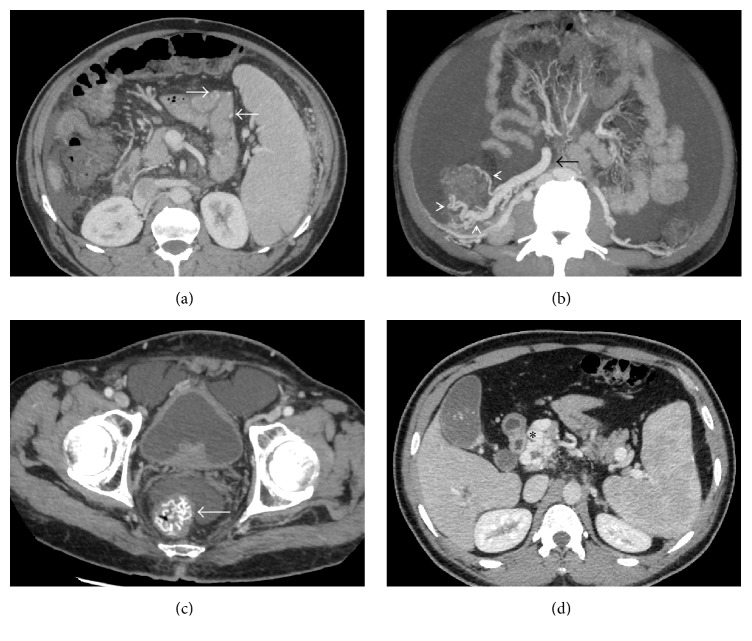
(a) Axial MIP image demonstrating multiple jejunal collaterals (arrows); (b) axial-oblique MIP CECT image showing multiple pericolonic collaterals (arrowheads) arising from the superior mesenteric vein (arrow); (c) axial MIP CECT image showing multiple rectal collaterals (arrow); (d) axial CECT image showing multiple paracholedochal collaterals (asterisks) encircling the common bile duct (arrow) in a patient with EHPVO.

**Table 1:** Common portosystemic collaterals [17–19].

Collaterals	Afferent	Efferent
*Common pathways*		

Esophageal varices	Left gastric vein	Azygos-hemiazygos veins

Gastric varices	Anterior branch of the left gastric vein (gastroesophageal varices Type 1) Short gastric and posterior gastric veins (gastroesophageal varices Type 2)	Esophageal or paraesophageal veins

Paraumbilical vein	Left portal vein	Anterior abdominal wall veins and iliofemoral veins

Perisplenic, splenoiliac	Splenic vein	Iliac vein

Internal-external mammary	Branches of portal veins, posterior or short gastric veins	Superior epigastric, inferior epigastric veins, and superficial veins of the thorax

Internal hemorrhoids	Inferior mesenteric vein	Iliac vein

Rectal varices	Superior rectal veins	Middle and inferior rectal veins, tributaries of internal iliac and pudendal veins

Gastrorenal shunt	Gastric varices or posterior or short gastric veins	Left renal vein

Splenorenal shunt	Splenic vein	Left renal vein

Pericholecystic varices	Cystic vein or a branch of the right portal vein	Hepatic vein, intrahepatic portal vein, or anterior abdominal wall collaterals

Mesenteric collaterals	Superior mesenteric vein and inferior mesenteric vein	Inferior vena cava through the retroperitoneal or pelvic veins (veins of Retzius)

Retroperitoneal collaterals	Colic or mesenteric branches (veins of Retzius)	Retrogastric varices or inferior phrenic veins to left renal vein or directly into the inferior vena cava

Omental varices	Superior or inferior mesenteric veins	Retroperitoneal or pelvic veins or gastroesophageal veins

*Uncommon pathways*		

Tracheal and bronchial varices	Tracheobronchial plexus of veins	Pulmonary veins Bronchial veins Esophageal/paraesophageal varices

Aberrant left gastric collateral	Left portal vein	Hepatogastric ligament

Colonic varices	Ileocolic, right, middle colic, or sigmoid colic vein	Right gonadal vein, right renal vein, and systemic lumbar veins

Jejunal or ileal varices	Jejunal and ileal veins	Abdominal wall veins or the veins of Retzius

Duodenal varices	Superior and inferior pancreaticoduodenal veins, cystic branches of the superior mesenteric veins, gastroduodenal vein, and pyloric vein	Veins of Retzius into the inferior vena cava

Pancreatic varices	Ventral and dorsal pancreatic veins, pancreaticoduodenal veins	Inferior vena cava

Uterovaginal varices	Superior hemorrhoidal plexus	Uterine and hypogastric veins to inferior vena cava

Vesical varices	Mesenteric veins (commonly root of mesentery)	Internal and external iliac veins

Bare area of the liver	Portal venous branches	Inferior phrenic and right internal thoracic vein

Vertebral collaterals	Innominate, vertebral, intercostal, lumbar, and sacral veins	Azygos and internal mammary pathways

Lateral thoracic	Lateral thoracic, thoracoepigastric, superficial circumflex, long saphenous, and femoral veins	Inferior vena cava

Sub-hepatic-porto-iliac	Main portal vein	Iliac veins

Gastrocaval shunt	Gastric varices or posterior gastric vein	Left inferior phrenic and pericardiophrenic vein to inferior vena cava

Transsplenic shunt	Splenic veins	Intercostal veins

Transhepatic shunt	Intrahepatic branches of the portal vein	Inferior vena cava, coronary vein, vertebral plexus, and hemiazygos vein

Right infradiaphragmatic shunt/apex type shunt	Peripheral branch of left portal vein	Internal thoracic vein and intercostal vein

Left infradiaphragmatic shunt/left triangular ligament shunt	Peripheral portal branch of the left lateral segment of liver	Intercostal vein or left pericardiophrenic vein to left inferior phrenic vein to inferior vena cava or left triangular ligament to inferior vena cava

Indirect gastrocaval shunt	Gastric varices or posterior or short gastric veins	Inferior phrenic vein

Mesentericorenal shunt	Mesenteric veins	Capsular renal veins or left renal vein

Mesenteric-gonadal shunt	Mesenteric veins	Right gonadal vein

Splenocaval/phrenic/ azygos shunt	Splenic vein or perisplenic collaterals	Hypogastric vein into inferior vena cava

**Table 2:** The Saad-Caldwell Classification of gastroesophageal varices.

Type 1	Isolated cardiogastric varices without fundic varices Type 1b – GRS + Type 1a – GRS –	Correlate with endoscopic Sarin classification: gastroesophageal varices Type 1 (GOV1)

Type 2	Isolated fundal gastric varices without cardiac extension Type 2b – GRS+ Type 2a – GRS –	Correlated endoscopic Sarin classification: isolated gastric varices Type 1 (IGV 1)

Type 3	High association with esophageal varices 3b – GRS + 3a – GRS –	Correlate with endoscopic Sarin classification: gastroesophageal varices Type 2 (GOV 2) Usually complex and large variceal systems

Type 4	Like Type 2 or Type 3 (more likely to be similar to Type 3)	Presence of splenic and/or portal venous thrombosis

**Table 3:** Differentiating features on endoscopy between hemorrhoids and rectal varices.

Rectal varices	Hemorrhoids
Extend superior to levator ani	Extension above levator ani not seen

Usually originate more than 4 cm above anal verge, distinct from hemorrhoids	Usually originated less than 4 cm below level of anal verge

Not contiguous with anal column or pectinate line	Maybe contiguous

Dilated, tortuous, submucosal vein, 3 to 6 mm in diameter and dark blue in color	Less dilated, nontortuous, paler, smaller in size

Do not prolapse into the proctoscope during examination	Prolapse into proctoscope commonly seen with higher grade
